# Indirect Comorbidity in Childhood and Adolescence

**DOI:** 10.3389/fpsyt.2013.00144

**Published:** 2013-11-04

**Authors:** William E. Copeland, Lilly Shanahan, Alaattin Erkanli, E. Jane Costello, Adrian Angold

**Affiliations:** ^1^Duke University, Durham, NC, USA; ^2^University of North Carolina at Chapel Hill, Chapel Hill, NC, USA

**Keywords:** comorbidity, epidemiology, oppositional defiant disorder, depression, attention-deficit/hyperactivity disorder

## Abstract

**Objective:** Comorbidity between psychiatric disorders is common, but pairwise associations between two disorders may be explained by the presence of other diagnoses that are associated with both disorders or “indirect” comorbidity.

**Materials and Methods:** Comorbidities of common childhood psychiatric disorders were tested in three community samples of children ages 6–17 (8931 observations of 2965 subjects). Psychiatric disorder status in all three samples was assessed with the Child and Adolescent Psychiatric Assessment. *Indirect* comorbidity was defined as A-B associations that decreased from significance to non-significance after adjusting for other disorders.

**Results:** All tested childhood psychiatric disorders were positively associated in bivariate analyses. After adjusting for comorbidities, many associations involving a behavioral disorder and an emotional disorder were attenuated suggesting indirect comorbidity. Generalized anxiety and depressive disorders displayed a very high level of overlap (adjusted OR = 37.9). All analyses were rerun with depressive disorders grouped with generalized anxiety disorder in a single “distress disorders” category. In these revised models, all associations between and emotional disorder and a behavior disorder met our criteria for indirect comorbidity except for the association of oppositional defiant disorder with distress disorders (OR = 11.3). Follow-up analyses suggested that the indirect associations were primarily accounted for by oppositional defiant disorder and the distress disorder category. There was little evidence of either sex differences or differences by developmental period.

**Conclusion:** After accounting for the overlap between depressive disorders with generalized anxiety disorder, direct comorbidity between emotional and behavioral disorders was uncommon. When there was evidence of indirect comorbidity, ODD, and distress disorders were the key intermediary diagnoses accounting for the apparent associations.

## Introduction

Numerous community studies [reviewed in (1)] using various versions of the ICD or DSM diagnostic systems and structured psychiatric interviews have documented the ubiquity of comorbidity among child and adolescent disorders [e.g., (2–10)]. These studies show that conduct disorder (CD), oppositional disorder (ODD), attention-deficit/hyperactivity disorder (ADHD), depressive disorders, and anxiety disorders co-occur more often than would be expected from their prevalences in the general population. The vast majority of studies to date have concentrated on pairwise, or occasionally three-way, diagnostic associations. Little is known, however, about which of these links persist after accounting for other comorbidities.

In a simplified scenario with just three disorders – anxiety, conduct, and depressive – all of which are significantly associated, it is possible that one of the three associations between disorders is merely a product of the other two: CD may be correlated with anxiety disorders only because both CD and anxiety disorders are correlated with depressive disorders. Thus, in this scenario, the apparent association (or comorbidity) is “indirect” and secondary to the direct associations with depressive disorders (1). This is an example of confounding (11) or of one disorder being a proxy for the other (12). The term “epiphenomenal” comorbidity (1) has been previously suggested, but some have been concerned that it could imply that such comorbidity was not “real.” We use that term “indirect” because it carries no such connotation.

The possibility of some comorbidities being indirect is important for a number of reasons. First, the apparent comorbidity of every disorder with all other disorders undermines the validity of the boundaries between diagnostic entities. Differential patterns of comorbidity would serve as evidence of divergent validity for the diagnostic system. Second, studying the “causes” of comorbidity between two disorders may be futile if, in fact, the observed link is indirect. If some combinations were found to be indirect, then the problem of identifying the causes of comorbidity would be simplified, since the number of combinations of interest would be smaller than the number of possible combinations. Third, identifying indirect comorbidity may help to resolve the question of why comorbidity is found between conditions with very disparate behavioral and emotional profiles. For instance, anxious and conduct disordered children lie at opposite ends of the reactivity spectrum (13–15), but the two disorders tend to co-occur.

Findings from the British Mental Health Survey of over 10,000 children ages 5–15 suggested that associations between ADHD and both anxiety and depression, CD and anxiety, and ODD and depression may be examples of indirect comorbidity (10). The authors concluded that “comorbidity is selective, being particularly evident between anxiety and depression, between ADHD and behavioral disorders, and between depression and at least some behavioral disorders (p. 1209).” An earlier analysis of sex-specific comorbidity patterns in the longitudinal Great Smoky Mountains Study (GSMS) study supported indirect associations of ADHD with depression and CD with anxiety, but found direct associations of ADHD and anxiety and ODD with depression (9).

Our aim here is to clarify which disorder associations are statistically independent and which associations attenuate after accounting for other disorders by combining data from three community samples with distinct racial and urban/rural configurations. Furthermore, this study will extend previous work by (1) treating common childhood anxiety disorders separately, rather than in aggregate (16, 17) and (2) grouping depressive disorders and generalized anxiety (18–20). Recent studies suggest ODD and irritability may play a key role in the associations between putative behavioral and emotional disorders (17, 21, 22). As such, we hypothesize that apparent associations between other putative behavioral disorders (CD and ADHD) and anxiety and depression may be indirect associations accounted for by ODD. As such, this study does not apply the hierarchical exclusion rules for diagnosing ODD only in the absence of CD.

A number of prior studies have look at the related issue of how common childhood disorders cluster, typically by using factor analysis (23–25). While these studies tell us which disorders are most strongly associated with one another, they do not clarify which disorders may account for apparent or indirect associations between pairs of disorder. These studies, however, converge with other evidence to support a strong association between depression and generalized anxiety with Lahey et al. (24) finding that “the latent factors of GAD and MDD were correlated near unity (p. 201).” Therefore, our analysis will test comorbidity patterns with the current DSM disorder structure and with also with depressive and generalized anxiety grouped into a single “distress disorders” category.

## Materials and Methods

### Sample and procedures

The current study uses data from three community-based studies of mental illness in childhood and adolescence that all used the same psychiatric interview. Together, the studies sample a racial/ethnically diverse group of children growing up in urban and rural settings (Table 1).

**Table 1:** **Characteristics of the three community studies**.

	Great smoky mountains study	Caring for children and the community	Diagnostic interview comparison study
Representative population sample?	Representative, rural	Representative, rural	Primary care, urban
Female	49.2%	49.9%	50.4%
Racial/Ethnicity	6.9% AA/89.6% WH	53.6% AA/41.1% WH	47.8% AA/43.9% WH
Number of data waves	7–10	3	1
Age range	8–16	9–17	9–16
Number of participants recruited at baseline	1420	920	627
Total observations	6674	1627	627
Assessment	Child and adolescent psychiatric assessment	Child and adolescent psychiatric assessment	Child and Adolescent Psychiatric Assessment

#### Great smoky mountains study

The GSMS is a longitudinal, representative study of children in 11 predominantly rural counties of North Carolina. Three cohorts of children, age 9, 11, and 13 years, were recruited from a pool of some 20,000 children using a two-stage sampling design, resulting in *N* = 1420 participants [49% female; see also (9)]. American Indians were oversampled to constitute 25% of the sample; seven percent of the participants were African American. Annual assessments were completed on the 1420 children until age 16 for a total of 6634 assessments. The youngest cohort was not interviewed at age 13 and half of the youngest cohort was interviewed at age 14 due to financial limitations. Sex-specific patterns of comorbidity have been presented in a prior publication (9).

#### Caring for children in the community

The CCC study is a longitudinal, representative study of 920 children aged 9–17 in four rural counties in North Carolina. The two-stage sampling design and methods are described in detail elsewhere (26). Briefly, a random sample of 17,117 9 to 17-year-olds in the public schools database generated a screening sample of 4500 youth. Of these, 3613 were contacted and agreed to complete screens (the externalizing scale of the CBCL). Of these families, 1302 were selected to participate in the interviews, and 920 (70.7%) interviews were completed. About 54% of the participants were African American and 50% were female. Two additional assessments were completed at 9 month intervals for subjects that had not yet reached age 18. Thus, there were 1627 assessments of 920 subjects.

#### Diagnostic interview comparison study

The DICS is study of 1261 children aged 9–16 recruited from an urban pediatric primary-care clinic to compare different structured diagnostic interviews (27). This present study includes the 630 children from DICS that were assessed with 3 month version of the Child and Adolescent Psychiatric Assessment (CAPA). In a normal year, half of the children attending the clinic were female, half were African American, one-third received Medicaid, Medicare, or other publicly funded health insurance, and 5% had no insurance coverage. Participants were recruited to provide equal numbers of children by sex, age (9–12 and 13–16), and race/ethnicity (white and non-white). Additional information on sampling, methods, and psychiatric prevalence rates for each study is available in prior publications [GSMS (9); CCC (26); DICS (27)].

For all studies, interviews were completed by the subject and a parent figure. Before interviews in each study began, parent and child signed informed consent/assent forms approved by the Duke University Medical Center Institutional Review Board. All interviewers had at least bachelor’s level degrees. They received 1 month of training and an audio recording of each interview was independently reviewed by a senior interviewer to insure that the interview was coded accurately.

### Measures

Psychiatric disorders in all samples were assessed using the same psychiatric interview: the CAPA (28, 29). Scoring programs for the CAPA, written in SAS (30) combined information about the date of onset, duration, and intensity of each symptom to create DSM-IV diagnoses and symptom scales. With the exception of ADHD, for which only parental reports were counted, a symptom was counted as present if it was reported by either the parent or the child, as is standard clinical practice. To minimize recall bias, the timeframe for determining the presence of most psychiatric symptoms was the preceding 3 months. Diagnostic groups included depressive disorders (including major depressive disorder, dysthymia, and minor depressive disorder), generalized anxiety disorder, social phobia, separation anxiety disorder, CD, ADHD, and ODD. Rare childhood disorders (prevalence less than 1%) were excluded from the current analysis (e.g., posttraumatic stress disorder, obsessive compulsive disorder, eating disorders). Two-week test-retest reliability of CAPA diagnoses in children aged 10–18 years is comparable to that of other highly structured interviews (*K*s for individual disorders range from 0.56 to 1.0) (28). The construct validity of CAPA diagnoses has been extensively supported (29).

### Analytic strategy

Rather than focusing on one outcomes variable, our goal was to model the associations between a set of dichotomous variables (diagnostic status) simultaneously. The general log-linear model, an extension of multiway frequency analysis, does not distinguish between independent and dependent variables, but focuses on mutual associations between variables (31, 32). This allows one to estimate patterns of dependence and independence among a set of variables. This approach was applied in SAS PROC GLIMMIX using the transition models discussed by Diggle et al. (33), and auto-logistic models described by Besag (34) to account for repeated observations. In the current analysis, bivariate unadjusted models were first tested between individual disorder pairs followed by a full multivariate adjusted model which accounted for all disorder associations simultaneously. Due to similarities across the different studies, models were fit using all three studies simultaneously (i.e., mega-analysis) with observations grouped within subject ID and subject ID grouped within study. Estimates from individual studies are available upon request.

Three general outcomes were possible. Unadjusted associations could be small (OR < 2.0) or non-significant suggesting no association and a lack of comorbidity. Disorder associations could continue to be significant and strong (OR ≥ 4.0) after accounting for other disorders – suggesting direct comorbidity. Third, strong, significant bivariate associations could attenuate to non-significance or small/moderate effects (2.0 ≤ OR < 4.0) in multivariable models, suggesting indirect comorbidity. Our goal was not to apply these standards and categories strictly, but with reasonable consideration of the size/significance of the adjusted odds ratio (OR) as well as the change in OR from the unadjusted model.

## Results

Prevalence rates of disorder groupings in each sample are provided in prior publications (9, 26, 27). Table 2 displays the unadjusted and adjusted associations between seven common childhood disorders with depressive disorders and generalized anxiety disorder treated separately. Un-shaded cells show associations among putative behavioral disorders (ADHD, CD, and oppositional defiant disorder). Cells shaded in dark gray show associations amongst putative emotional disorders (depressive disorders, generalized anxiety, social phobia, and separation anxiety). Cells shaded in light gray show associations between emotional and behavioral disorders.

**Table 2:** **Unadjusted and adjusted pairwise comorbidity for common childhood diagnoses**.

	CD	ODD	ADHD	DEP	GAD	SOC

	OR (95% CI)	OR (95% CI)	OR (95% CI)	OR (95% CI)	OR (95% CI)	OR (95% CI)
ODD						
Unadjusted	**16.1 (11.0–23.5)**^‡^					
Adjusted	**11.5 (7.5**–**17.5)**^‡^					
ADHD
Unadjusted	**7.5 (4.9**–**11.3)**^‡^	**12.9 (9.2**–**18.3)**^‡^				
Adjusted	**2.4 (1.5**–**3.8)**^‡^	**6.1 (3.8**–**9.8)**^‡^				
DEP
Unadjusted	**7.2 (4.7**–**11.1)**^‡^	**19.3 (13.8**–**27.1)**^‡^	**8.0 (5.2**–**12.4)**^‡^			
Adjusted	**2.5 (1.3**–5.0)*	**10.9 (6.1**–**19.4)**^‡^	1.5 (0.8–3.0)			
GAD
Unadjusted	**4.1 (2.2**–**7.7)**^‡^	**12.3 (7.2**–**21.1)**^‡^	**10.9 (6.1**–**19.3)**^‡^	**54.1 (33.9**–**86.3)**^‡^		
Adjusted	0.6 (0.3–1.4)	1.8 (0.7–4.3)	2.0 (0.8–4.5)	**37.9 (7.9**–**80.5)**^‡^		
SOC
Unadjusted	**2.9 (1.4**–6.0)*	**8.6 (4.9**–**14.8)**^‡^	**10.7 (5.7**–**20.0)**^‡^	**20.7 (10.8**–**39.8)**^‡^	**20.0 (10.1**–**39.6)**^‡^	
Adjusted	0.6 (0.2–1.5)	1.9 (0.9–3.9)	**3.4 (1.4**–8.2)*	**9.9 (4.3**–**22.9)**^‡^	2.1 (0.9–5.1)	
SEP
Unadjusted	**3.7 (2.2**–**6.4)**^‡^	**6.2 (4.0**–**9.9)**^‡^	**9.2 (5.5**–**15.2)**^‡^	**7.5 (4.6**–**12.2)**^‡^	**19.6 (11.2**–**34.4)**^‡^	**13.9 (7.6**–**25.3)**^‡^
Adjusted	1.7 (0.9–3.2)	**2.2 (1.1**–**4.1)**	**3.3 (1.7**–**6.4)**^‡^	1.4 (0.6–3.2)	**8.1 (2.9**–**22.6)**^‡^	**5.1 (2.3**–**11.2)**^‡^

CD, conduct disorder; DEP, depressive disorders; ODD, oppositional defiant disorder; ADHD, attention-deficit/hyperactivity disorder; GAD, generalized anxiety disorder; SOC, social phobia; SEP, separation anxiety disorders.

Odds ratios in bold are significant at the p < 0.05 level. *p < 0.01. ^‡^p < 0.001.

All 21 unadjusted pairwise associations were positive and statistically significant and 19 were large effects (OR ≥ 4.0). The adjusted model provides pairwise associations after accounting for comorbidities. Of the significant pairwise associations, nine were no longer statistically associated in the adjusted model and others were attenuated to small/moderate effects. Many of these involved an association between an emotional disorder and a behavioral disorder (e.g., ADHD and depressive disorder or CD with social phobia), but there was also evidence of associations between behavioral or emotional disorders that were attenuated (e.g., GAD and social phobia, ADHD and CD). A number of these attenuated associations involved either depressive disorders or generalized anxiety. The adjusted association between depression and generalized anxiety, however, was very high with an OR of 37.9. This high level of overlap may adversely affect the accuracy of individual associations within a multivariate model with associations with one disorder very high and with the overlapping disorder very low (11).

The higher overlap between depression and generalized anxiety is consistent with prior studies suggesting that the association between generalized anxiety and depression is sufficiently strong to recommend grouping these categories. Therefore, we reran the adjusted model with generalized anxiety and depressive disorders grouped in one category as distress disorders (Table 3). All unadjusted pairwise associations continued to be significant and all but 1 were strong effects (exception was CD and separation anxiety).

**Table 3:** **Unadjusted and adjusted pairwise comorbidity for common childhood diagnoses with generalized anxiety and depression grouped together**.

	CD	ODD	ADHD	DD	SOC

	OR (95% CI)	OR (95% CI)	OR (95% CI)	OR (95% CI)	OR (95% CI)
ODD
Unadjusted	**16.1 (11.0**–**23.5)**^‡^				
Adjusted	**11.3 (7.4**–**17.3)**^‡^				
ADHD
Unadjusted	**7.5 (4.9**–**11.3)**^‡^	**12.9 (9.2**–**18.3)**^‡^			
Adjusted	**2.4 (1.5**–**3.7)**^‡^	**6.0 (3.7**–**9.6)**^‡^			
DD
Unadjusted	**7.0 (4.6**–**10.4)**^‡^	**18.4 (13.5**–**25.2)**^‡^	**8.5 (5.7**–**12.7)**^‡^		
Adjusted	**2.3 (1.3**–4)*	**11.3 (7.1**–**18)**^‡^	**2.1 (1.1**–**3.7)**		
SOC
Unadjusted	**4.1 (2.2**–**7.7)**^‡^	**12.3 (7.2**–**21.1)**^‡^	**10.9 (6.1**–**19.3)**^‡^	**20.6 (11.1**–**38.3)**^‡^	
Adjusted	0.6 (0.2–1.5)	**2.0 (1.0**–**4.1)**	**3.4 (1.5**–8.0)*	**11.6 (5.2**–**25.8)**^‡^	
SEP					
Unadjusted	**2.9 (1.4**–6.0)*	**8.6 (4.9**–**14.8)**^‡^	**10.7 (5.7**–**20.0)**^‡^	**11.0 (7.1**–**17.1)**^‡^	**20.0 (10.1**–**39.6)**^‡^
Adjusted	1.4 (0.8–2.8)	1.8 (0.9–3.4)	**3.3 (1.8**–**6.2)**^‡^	**5.7 (3.0**–**10.8)**^‡^	**4.3 (2**–**9.3)**^‡^

CD, conduct disorder; DD, distress disorders; ODD, oppositional defiant disorder; ADHD, attention-deficit/hyperactivity disorder; GAD, generalized anxiety disorder; SOC, social phobia; SEP, separation anxiety disorders.

Odds ratios in bold are significant at the *p* < 0.05 level. *p < 0.01. ^‡^*p* < 0.001.

Almost all adjusted associations between emotional disorders and behavioral disorders were either non-significant or attenuated to small/moderate effects, thus meeting our criterion for indirect comorbidity. The lone exception was the association of the distress disorders category with ODD which was still strong suggesting a direct association. The association of CD with social phobia was the only instance in which an adjusted association had an OR of less than 1.0. The only instance of indirect comorbidity between like disorders involved associations that were attenuated but still statistically significant (CD and ADHD).

### Follow-up analyses

**Figure 1:**
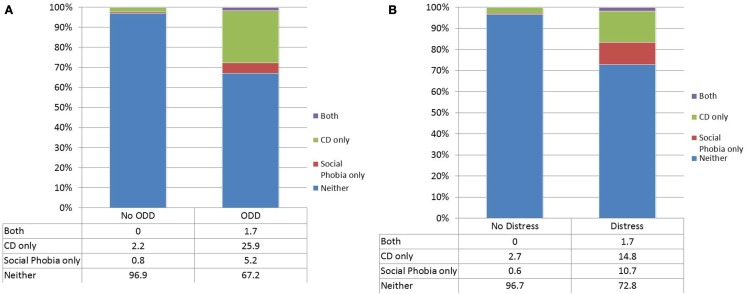
**The overlap between conduct disorder and social phobia as a function of either oppositional defiant disorder (A) or the distress disorder category (B)**.

**Figure 2:**
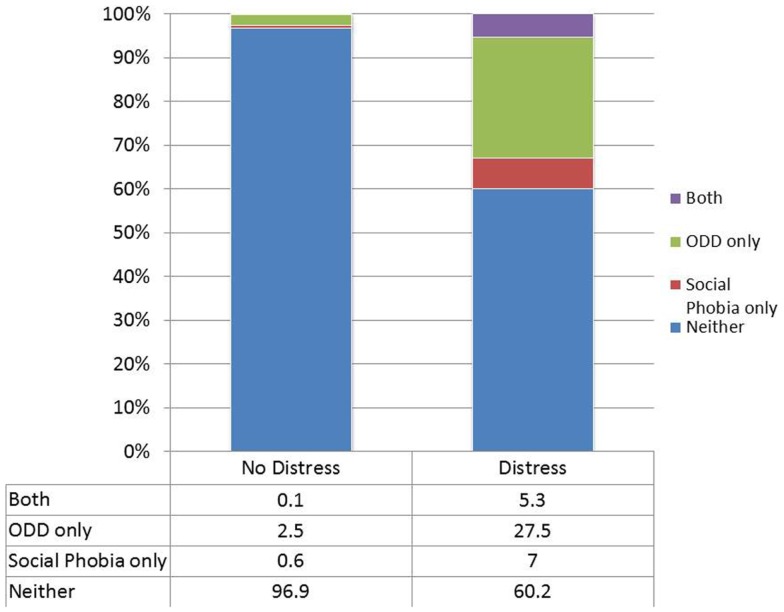
**The overlap between oppositional defiant disorder and social phobia as a function of the distress disorder category**.

We followed up on each specific instances of indirect comorbidity to determine which disorders attenuated the unadjusted associations. In the case of CD with social phobia, the association was attenuated by the inclusion of either distress disorder or oppositional defiant disorder. Figure 1 shows the overlap between CD and social phobia in those with or without oppositional defiant disorder. Almost all of the overlap occurred in those with oppositional defiant disorder. Figure 2 shows the overlap between CD and social phobia as a function of distress disorder status. In the case of oppositional defiant disorder and social phobia, the association was attenuated by inclusion of distress disorders (Figure 2).

Finally, we tested whether adjusted associations differed by sex or developmental period (childhood vs. adolescence). The variable for developmental period grouped observations from ages 9 to 13 and 14 to 16 as this corresponds to age-related changes in prevalence of various childhood disorders (9). None of the adjusted comorbidity estimates were moderated by sex and only one was moderated by developmental period: the association of CD with social phobia. The adjusted association was not significant in the earlier age group (OR = 1.2 95% CI: 0.5–3.6, *p* = 0.63) and then shifted to a significant negative association in adolescence (OR = 0.1 95% CI: 0.0–1.0, *p* = 0.05). This is the only pairwise association to display a significant inverse association.

## Discussion

In simple bivariate analyses, comorbidity appeared to be the rule: Each childhood disorder was associated with increased risk for every other disorder, albeit to varying degrees. This pattern of universal comorbidity is precisely the pattern expected based upon a meta-analysis of comorbidity patterns in childhood and adolescence (1). At the same time, it is not at all clear why disorders as different as CD and anxiety disorders should co-occur.

The question for the current study was whether there were certain pairwise associations where the apparent association was the result of other disorders with which both disorders co-occurred. Generalized anxiety and depressive disorders overlapped so much that it made sense to group these disorders as has been suggested by others (18). With this grouping, direct comorbidity was uncommon. All associations association of a behavioral disorder with an emotional disorder met criteria for indirect comorbidity. The lone exception to this pattern was the direct comorbidity of ODD with distress disorders.

One of the longstanding contradictions of comorbidity research has been the persistent evidence that disorders that lie at opposite ends of the reactivity spectrum (e.g., CD and anxiety disorders) commonly co-occur (1). This study suggests that such associations are accounted for by the proxy risk factors of oppositional defiant disorder or the distress disorder category. Factor analytic studies have helped to clarify how disorders tend to cluster (24, 25), and such studies have tended to support DSM-based distinctions while recommending a category that combines major depressive disorder and generalized anxiety. This analysis, however, clarifies the apparent overlap between putative externalizing and internalizing disorders by identifying the role of proxy risk factors (12). Previous studies have either suggested that the association between CD and anxiety is indirect (10) or observed a lack of association (35, 36). These studies and others looking at comorbidities have typically aggregated multiple anxiety disorders and combined CD and ODD into a single group (1). Findings from our study do not support this approach, and patterns of comorbidity differed in part for CD and oppositional defiant disorder. For example, CD was not associated with social phobia in the adjusted models but oppositional defiant disorder continued to have a modest positive association. It may be unnecessary, therefore, to pursue any explanation for the co-occurrence of either CD with select individual anxiety disorders beyond the joint associations of CD and anxiety disorders with oppositional defiant disorder or distress disorder. Others have also found similar evidence of indirect comorbidity between ADHD and depression (10) or simply noted relative weak pairwise associations (4, 7). Our analysis supports this conclusion.

Our results showed difference between ODD and CD in their associations with other disorders (e.g., ADHD, distress disorders). This serves to inform meta-analytic findings suggesting strong associations of CD/ODD with ADHD and depression (1). The meta-analysis was limited by the convention in many studies of combining CD with ODD. It is easy to presume that the putatively “more severe” CD is driving the associations, but this study suggests that this not the case and that ODD may account for much of the associations between ADHD and depression with CD. This contradicts findings in clinical samples that children with ADHD and CD represent a distinct subtype [e.g., (37)] or that ODD is a subsyndromal form of CD (38). This assumption is similarly misguided in looking at prediction from childhood to young adulthood where ODD, not CD, predicts later anxiety and depressive disorders (17). Together, this evidence suggests that there are problems with both the ICD-10 practice of placing CD and ODD together in a single diagnosis of CD, with “ODD” as a subtype (39) and the DSM-IV exclusionary criterion for ODD which only allows the diagnosis if the criteria for CD are not met. Perhaps more importantly, our study emphasizes the relationship between ODD and depression/generalized anxiety as substantially responsible for driving the association between distinct emotional and behavioral disorders.

This, in turn, leads to the question of why the relationship between ODD and depression/generalized anxiety (or distress disorders) should be so strong. A prime suspect must surely be the role of irritability. Factor analyses of the symptoms of ODD reliably yield an irritability factor, and irritable mood is common in depression (and included in the diagnostic criteria for children and adolescents) (21). Perhaps it is irritability that provides the key link between the emotional and disruptive disorders. ODD and depression (or distress disorders) sit in the middle of the web of associations between emotional and behavioral disorders. This observation informed the inclusion of Disruptive Mood Dysregulation Disorder in DSM 5.0 to capture children with persistent negative mood coupled with frequent behavioral outbursts (40). This disorder seems to do a good job of accounting for severe, non-episodic irritability (41), but still leaves questions about the more general of irritability in common childhood psychiatric disorders.

Despite findings of indirect comorbidity, it is important to be clear that the unadjusted associations indicate that these disorders do indeed often co-occur. That apparent co-occurrence, however, is often explained by comorbidity of both disorders with an intermediary disorder(s). This observation affects a significant literature that has attempted to understand within-disorder heterogeneity by parsing different comorbid subgroups [e.g., (42, 43)]. This study suggests that the number of such comorbid subgroups that merit study is more limited than that of all possible combinations. This is not a trivial concern as comorbidity research for the past 20 years has consistently reported positive, significant associations between CD and anxiety (1).

### Caveats

This study benefits from using three community samples with distinct urban/rural and racial/ethnic characteristics that all used the same diagnostic interview. These samples generate different prevalence rates for common psychiatric disorders; nevertheless, there was remarkable convergence across studies in terms patterns of comorbidity, such that we were able to conduct a single analysis across all three samples. This provides a robust set of estimates and finding that are independent of individual study characteristics. None of the studies included, however, is nationally representative. Our results cannot be generalized to children below the age of nine. All three studies focused on *recent* symptoms in the past three months to minimize forgetting or recall bias. At the same time, it is entirely possible that subjects may have met criteria for a disorder order prior to involvement in the study or between assessments.

### Clinical implications

It has long been observed that common psychiatric disorders are more likely to co-occur than would be expected by chance. Comorbidity is the rule in psychiatric research in epidemiological samples, and there is extensive evidence that such co-occurrence is even more common in the clinical setting (44, 45). Knowledge of indirect comorbidity may allow the clinician to avoid diagnostic misdirection. The role of the clinician is to discriminate the information that might guide accurate prognosis and effective treatment from the wealth of information presented by the client. Understanding how common disorders co-occur and which disorders account for *apparent* co-occurrence may be a key step in differential diagnosis and treatment prioritization.

## Conflict of Interest Statement

The authors declare that the research was conducted in the absence of any commercial or financial relationships that could be construed as a potential conflict of interest.
